# Supplementary material for: Geographical, temporal and individual factors influencing foraging behaviour and consistency in Australasian gannets
Source: R Soc Open Sci. 2020 May 27;7(5):181423. doi: 10.1098/rsos.181423 (PMC7277272; doi:10.1098/rsos.181423)
Supplement: Model selection results [file rsos181423supp1.pdf]

Geographic, temporal and individual factors influencing foraging behaviour and consistency in Australasian gannets

Marlenne A. Rodríguez-Malagón[1], Elodie C.M. Camprasse[1], Lauren P. Angel[1] and John P.Y. Arnould*[1]

[1]Deakin University, Geelong, Australia. School of Life and Environmental Sciences (Burwood Campus)

*Corresponding author: john.arnould@deakin.edu.au, +61(0) 438 212 334

SUPPORTING INFORMATION

Table S1 AICc based model selection (Δ < 4) for factors that influence the foraging metrics in adult free-ranging Australasian gannets (*Morus serrator*). Model variables: BCI: Body Condition Index; BSI: Body Size Index; WLI: Wing Length Index; cln: colony; stage: breeding stage; yr: year; sex

Foraging metric	Model fixed effects	df	AICc	ΔAIC	AIC Weight
Maximum distance from the colony (km)	WLI + cln + stage + yr + sex	12	6723.60	0	0.24
	cln + stage + yr + sex	11	6724.10	0.50	0.19
	BCI + cln + stage + yr + sex	12	6724.50	0.92	0.15
	BCI + WLI + cln + stage + yr + sex	13	6724.80	1.25	0.13
	BSI + WLI + cln + stage + yr + sex	13	6725.60	2.02	0.09
	BSI + cln + stage + yr + sex	12	6726.00	2.44	0.07
	BCI + BSI + cln + stage + yr + sex	13	6726.50	2.91	0.06
	BCI + BSI + WLI + cln + stage + yr + sex	14	6726.80	3.27	0.05
Bearing (°)	BSI + cln + stage + yr	8	27346.30	0	0.16
	cln + stage + yr	7	27346.50	0.25	0.14

	BCI + cln + stage + yr	8	27347.10	0.80	0.11
	BCI + BSI + cln + stage + yr	9	27347.10	0.83	0.11
	BSI + WLI + cln + stage + yr	9	27348.20	1.89	0.06
	WLI + cln + stage + yr	8	27348.20	1.92	0.06
	BSI + cln + stage + yr + sex	9	27348.20	1.98	0.06
	cln + stage + yr + sex	8	27348.50	2.23	0.05
	BCI + WLI + cln + stage + yr	9	27349.00	2.74	0.04
	BCI + cln + stage + yr + sex	9	27349.00	2.78	0.04
	BCI + BSI + cln + stage + yr + sex	10	27349.10	2.82	0.04
	BCI + BSI + WLI + cln + stage + yr	10	27349.10	2.84	0.04
	BSI + WLI + cln + stage + yr + sex	10	27350.10	3.88	0.02
	WLI + cln + stage + yr + sex	9	27350.20	3.91	0.02
Tortuosity Index	cln + stage + yr	7	-5741.90	0	0.20
	WLI + cln + stage + yr	8	-5741.70	0.28	0.18
	BCI + cln + stage + yr	8	-5740.20	1.79	0.08
	cln + stage + yr + sex	8	-5740.00	1.95	0.08
	BSI + cln + stage + yr	8	-5739.90	2.01	0.08
	WLI + cln + stage + yr + sex	9	-5739.70	2.22	0.07
	BSI + WLI + cln + stage + yr	9	-5739.70	2.25	0.07
	BCI + WLI + cln + stage + yr	9	-5739.70	2.28	0.07
	BCI + cln + stage + yr + sex	9	-5738.20	3.74	0.03
	BCI + BSI + cln + stage + yr	9	-5738.10	3.80	0.03
	BSI + cln + stage + yr + sex	9	-5738.00	3.96	0.03
Mean VeDBA	stage + sex	7	-2754	0	0.14
	cln + stage + sex	8	-2753.9	0.14	0.13
	stage + year + sex	8	-2752.1	1.93	0.05
	stage + BSI + sex	8	-2752	2.02	0.05
	stage + WLI + sex	8	-2752	2.02	0.05
	BCI + stage + sex	8	-2752	2.02	0.05
	cln + stage + year + sex	9	-2751.9	2.06	0.05
	BCI + cln + stage + sex	9	-2751.9	2.12	0.05
	cln + stage + BSI + sex	9	-2751.8	2.16	0.05

	cln + stage + WLI + sex	9	-2751.8	2.17	0.05
	stage + BSI + year + sex	9	-2750.1	3.94	0.02
	BCI + stage + year + sex	9	-2750	3.95	0.02
	stage + WLI + year + sex	9	-2750	3.96	0.02
Number of dives	stage + sex	6	5068.70	0	0.09
	stage + yr + sex	7	5068.80	0.09	0.08
	stage	5	5069.90	1.2	0.05
	stage + yr	6	5070.20	1.46	0.04
	BSI + stage + sex	7	5070.20	1.49	0.04
	WLI + stage + sex	7	5070.70	1.99	0.03
	BCI + stage + sex	7	5070.80	2.02	0.03
	cln + stage + sex	7	5070.80	2.02	0.03
	WLI + stage + yr + sex	8	5070.80	2.04	0.03
	BSI + stage + yr + sex	8	5070.80	2.09	0.03
	BCI + stage + yr + sex	8	5070.90	2.11	0.03
	cln + stage + yr + sex	8	5070.90	2.11	0.03
	BSI + stage	6	5071.40	2.7	0.02
	WLI + stage	6	5071.90	3.18	0.02
	BCI + stage	6	5072.00	3.22	0.02
	cln + stage	6	5072.00	3.22	0.02
	WLI + stage + yr	7	5072.20	3.42	0.02
	BSI + stage + yr	7	5072.20	3.46	0.02
	BCI + stage + yr	7	5072.20	3.49	0.02
	cln + stage + yr	7	5072.20	3.49	0.02
	BCI + BSI + stage + sex	8	5072.30	3.51	0.02
	BSI + cln + stage + sex	8	5072.30	3.51	0.02
	BSI + WLI + stage + sex	8	5072.30	3.51	0.02

Table S2 Average model coefficients and relative importance of variables included in top model set (ΔAICc ≤ 4) explaining individual variation in Australasian gannets (*Morus serrator*) foraging metrics

Foraging metric	Parameter	Estimate	SE	5 % CI	95 % CI	Relative importance
Maximum distance from the colony (km)	(Intercept)	4.06	0.40	3.42	4.72	-
	Colony (PE)	-0.81	0.09	-0.96	-0.66	1.00
	Breeding stage (INC)	0.51	0.06	0.41	0.62	1.00
	Breeding stage (LCR)	-0.21	0.06	-0.31	-0.11	1.00
	Year (2015)	0.14	0.05	0.06	0.21	1.00
	Sex (male)	-0.33	0.09	-0.48	-0.18	1.00
	WLI	0.02	0.01	-0.01	0.03	0.52
	BCI	0.05	0.05	-0.02	0.12	0.40
	BSI	0.01	0.01	-0.01	0.01	0.27
Bearing (º)	(Intercept)	213.12	27.04	167.93	257.68	-
	Colony (PE)	-72.44	6.23	-82.76	-62.13	1.00
	Breeding stage (INC)	-16.96	3.99	-23.52	-10.40	1.00
	Breeding stage (LCR)	11.74	3.82	5.45	18.03	1.00
	Year (2015)	13.69	3.32	8.19	19.12	1.00
	BSI	0.01	0.01	-0.01	0.03	0.51
	BCI	3.38	2.99	-1.55	8.30	0.39
	WLI	0.26	0.73	-0.95	1.45	0.26
	Sex (male)	1.06	6.18	-9.16	11.30	0.25
Tortuosity Index	(Intercept)	0.31	0.02	0.28	0.33	-
	Colony (PE)	-0.05	0.01	-0.06	-0.04	1.00
	Breeding stage (INC)	-0.05	0.01	-0.06	-0.04	1.00
	Breeding stage (LCR)	-0.01	0.01	-0.01	0.01	1.00
	Year (2015)	0.04	0.01	0.04	0.05	1.00
	WLI	0.01	0.01	-0.01	0.01	0.42
	BCI	0.01	0.01	-0.01	0.01	0.23
	Sex (male)	-0.01	0.01	-0.01	0.01	0.23
	BSI	-0.01	0.01	-0.01	0.01	0.22
Mean VeDBA	(Intercept)	0.83	0.02	0.79	0.86	-

	Breeding stage (INC)	-0.04	0.01	-0.05	-0.03	1.00
	Breeding stage (LCR)	0.01	0.01	-0.01	0.01	1.00
	Sex (male)	0.03	0.01	0.02	0.04	1.00
	Colony (PE)	0.01	0.01	-0.00	0.02	0.44
	Year (2015)	-0.01	0.01	-0.01	0.01	0.22
	BSI	0.01	0.01	-0.01	0.01	0.16
	WLI	0.01	0.01	-0.01	0.01	0.16
	BCI	-0.01	0.01	-0.01	0.01	0.16
Number of dives	(Intercept)	6.63	0.43	5.77	7.48	-
	Breeding stage (INC)	1.53	0.18	1.24	1.83	1.00
	Breeding stage (LCR)	0.28	0.15	0.02	0.53	1.00
	Sex (male)	-0.33	0.18	-0.63	-0.03	0.68
	Year (2015)	-0.21	0.16	-0.47	0.05	0.44
	BSI	-0.01	0.01	-0.01	0.01	0.22
	WLI	-0.01	0.02	-0.04	0.03	0.16
	BCI	-0.01	0.09	-0.15	0.15	0.16
	Colony (PE)	0.01	0.18	-0.30	0.30	0.16

Table S3 Factors influencing individual variation (measured as the coefficient of variation within deployments for each foraging metric), at short- term scale (T-to-T) in Australasian gannets (*Morus serrator*). Most parsimonious models after model averaging and their corresponding estimated regression parameters are shown. The most parsimonious model was selected with using Akaike information criterion (AICc, Δ < 4). Model variables: BCI: Body Condition Index; BSI: Body Size Index; WLI: Wing Length Index; stage: breeding stage; colony; year; sex

Response	Most parsimonious model	Fixed effect	Estimate	SE	*t*-value	*P*-value
Distance from colony (km)*	colony + year	(Intercept)	0.28	0.01	24.32	<0.0001
		Colony (PE)	-0.05	0.01	-4.49	<0.0001
		Year (2015)	-0.06	0.01	-4.25	<0.0001
Bearing (º)	stage + year	(Intercept)	1.60	0.04	37.19	<0.0001
		Stage (INC)	-0.26	0.06	-4.24	<0.0001
		Stage (LCR)	0.07	0.06	1.10	0.27
		Year 2015	0.11	0.05	2.06	0.04
Tortuosity index	colony + WLI + year + sex	(Intercept)	0.22	0.03	22.26	<0.0001
		Colony (PE)	0.04	0.01	3.68	<0.0001
		Year (2015)	-0.04	0.01	-3.96	<0.0001
		Sex (male)	0.02	0.01	2.54	0.01
		WLI	-0.01	0.01	-2.70	0.01
		Sex (male)	0.03	0.01	1.83	0.07
Mean VeDBA (g)*	BSI	(Intercept)	0.08	0.01	21.82	<0.0001

		BSI	0.01	0.01	2.70	0.01
Number of dives*	colony + stage + year	(Intercept)	0.25	0.01	16.92	<0.0001
		Colony (PE)	-0.04	0.01	-2.66	0.01
		Year (2015)	-0.06	0.02	-3.98	0.00
		Stage (INC)	0.01	0.02	0.35	0.73
		Stage (LCR)	0.06	0.02	3.57	0.001

*Transformed variable
