# Supplementary material for: Geographical, temporal and individual factors influencing foraging behaviour and consistency in Australasian gannets
Source: R Soc Open Sci. 2020 May 27;7(5):181423. doi: 10.1098/rsos.181423 (PMC7277272; doi:10.1098/rsos.181423)
Supplement: Model selection results [file rsos181423supp2.zip › Supplementary Tables/Table S1 - RSOS-181423.R2.docx]

**Table S1** AICc based model selection (∆ < 4) for factors that influence the foraging metrics in adult free-ranging Australasian gannets (*Morus serrator*). Model variables: BCI: Body Condition Index; BSI: Body Size Index; WLI: Wing Length Index; cln: colony; stage: breeding stage; yr: year; sex

| Foraging  metric | Model fixed effects | df | AICc | ∆AIC | AIC  Weight |
| --- | --- | --- | --- | --- | --- |
| Maximum distance from the colony  (km) | WLI + cln + stage + yr + sex | 12 | 6723.60 | 0 | 0.24 |
|  | cln + stage + yr + sex | 11 | 6724.10 | 0.50 | 0.19 |
|  | BCI + cln + stage + yr + sex | 12 | 6724.50 | 0.92 | 0.15 |
|  | BCI + WLI + cln + stage + yr + sex | 13 | 6724.80 | 1.25 | 0.13 |
|  | BSI + WLI + cln + stage + yr + sex | 13 | 6725.60 | 2.02 | 0.09 |
|  | BSI + cln + stage + yr + sex | 12 | 6726.00 | 2.44 | 0.07 |
|  | BCI + BSI + cln + stage + yr + sex | 13 | 6726.50 | 2.91 | 0.06 |
|  | BCI + BSI + WLI + cln + stage + yr + sex | 14 | 6726.80 | 3.27 | 0.05 |
| Bearing (º) | BSI + cln + stage + yr | 8 | 27346.30 | 0 | 0.16 |
|  | cln + stage + yr | 7 | 27346.50 | 0.25 | 0.14 |

|  | BCI + cln + stage + yr | 8 | 27347.10 | 0.80 | 0.11 |
| --- | --- | --- | --- | --- | --- |
|  | BCI + BSI + cln + stage + yr | 9 | 27347.10 | 0.83 | 0.11 |
|  | BSI + WLI + cln + stage + yr | 9 | 27348.20 | 1.89 | 0.06 |
|  | WLI + cln + stage + yr | 8 | 27348.20 | 1.92 | 0.06 |
|  | BSI + cln + stage + yr + sex | 9 | 27348.20 | 1.98 | 0.06 |
|  | cln + stage + yr + sex | 8 | 27348.50 | 2.23 | 0.05 |
|  | BCI + WLI + cln + stage + yr | 9 | 27349.00 | 2.74 | 0.04 |
|  | BCI + cln + stage + yr + sex | 9 | 27349.00 | 2.78 | 0.04 |
|  | BCI + BSI + cln + stage + yr + sex | 10 | 27349.10 | 2.82 | 0.04 |
|  | BCI + BSI + WLI + cln + stage + yr | 10 | 27349.10 | 2.84 | 0.04 |
|  | BSI + WLI + cln + stage + yr + sex | 10 | 27350.10 | 3.88 | 0.02 |
|  | WLI + cln + stage + yr + sex | 9 | 27350.20 | 3.91 | 0.02 |
| Tortuosity  Index | cln + stage + yr | 7 | -5741.90 | 0 | 0.20 |
|  | WLI + cln + stage + yr | 8 | -5741.70 | 0.28 | 0.18 |
|  | BCI + cln + stage + yr | 8 | -5740.20 | 1.79 | 0.08 |
|  | cln + stage + yr + sex | 8 | -5740.00 | 1.95 | 0.08 |
|  | BSI + cln + stage + yr | 8 | -5739.90 | 2.01 | 0.08 |
|  | WLI + cln + stage + yr + sex | 9 | -5739.70 | 2.22 | 0.07 |
|  | BSI + WLI + cln + stage + yr | 9 | -5739.70 | 2.25 | 0.07 |
|  | BCI + WLI + cln + stage + yr | 9 | -5739.70 | 2.28 | 0.07 |
|  | BCI + cln + stage + yr + sex | 9 | -5738.20 | 3.74 | 0.03 |
|  | BCI + BSI + cln + stage + yr | 9 | -5738.10 | 3.80 | 0.03 |
|  | BSI + cln + stage + yr + sex | 9 | -5738.00 | 3.96 | 0.03 |
| Mean  VeDBA | stage + sex | 7 | -2754 | 0 | 0.14 |
|  | cln + stage + sex | 8 | -2753.9 | 0.14 | 0.13 |
|  | stage + year + sex | 8 | -2752.1 | 1.93 | 0.05 |
|  | stage + BSI + sex | 8 | -2752 | 2.02 | 0.05 |
|  | stage + WLI + sex | 8 | -2752 | 2.02 | 0.05 |
|  | BCI + stage + sex | 8 | -2752 | 2.02 | 0.05 |
|  | cln + stage + year + sex | 9 | -2751.9 | 2.06 | 0.05 |
|  | BCI + cln + stage + sex | 9 | -2751.9 | 2.12 | 0.05 |
|  | cln + stage + BSI + sex | 9 | -2751.8 | 2.16 | 0.05 |

|  | cln + stage + WLI + sex | 9 | -2751.8 | 2.17 | 0.05 |
| --- | --- | --- | --- | --- | --- |
|  | stage + BSI + year + sex | 9 | -2750.1 | 3.94 | 0.02 |
|  | BCI + stage + year + sex | 9 | -2750 | 3.95 | 0.02 |
|  | stage + WLI + year + sex | 9 | -2750 | 3.96 | 0.02 |
| Number of  dives | stage + sex | 6 | 5068.70 | 0 | 0.09 |
|  | stage + yr + sex | 7 | 5068.80 | 0.09 | 0.08 |
|  | stage | 5 | 5069.90 | 1.2 | 0.05 |
|  | stage + yr | 6 | 5070.20 | 1.46 | 0.04 |
|  | BSI + stage + sex | 7 | 5070.20 | 1.49 | 0.04 |
|  | WLI + stage + sex | 7 | 5070.70 | 1.99 | 0.03 |
|  | BCI + stage + sex | 7 | 5070.80 | 2.02 | 0.03 |
|  | cln + stage + sex | 7 | 5070.80 | 2.02 | 0.03 |
|  | WLI + stage + yr + sex | 8 | 5070.80 | 2.04 | 0.03 |
|  | BSI + stage + yr + sex | 8 | 5070.80 | 2.09 | 0.03 |
|  | BCI + stage + yr + sex | 8 | 5070.90 | 2.11 | 0.03 |
|  | cln + stage + yr + sex | 8 | 5070.90 | 2.11 | 0.03 |
|  | BSI + stage | 6 | 5071.40 | 2.7 | 0.02 |
|  | WLI + stage | 6 | 5071.90 | 3.18 | 0.02 |
|  | BCI + stage | 6 | 5072.00 | 3.22 | 0.02 |
|  | cln + stage | 6 | 5072.00 | 3.22 | 0.02 |
|  | WLI + stage + yr | 7 | 5072.20 | 3.42 | 0.02 |
|  | BSI + stage + yr | 7 | 5072.20 | 3.46 | 0.02 |
|  | BCI + stage + yr | 7 | 5072.20 | 3.49 | 0.02 |
|  | cln + stage + yr | 7 | 5072.20 | 3.49 | 0.02 |
|  | BCI + BSI + stage + sex | 8 | 5072.30 | 3.51 | 0.02 |
|  | BSI + cln + stage + sex | 8 | 5072.30 | 3.51 | 0.02 |
|  | BSI + WLI + stage + sex | 8 | 5072.30 | 3.51 | 0.02 |
