# Supplementary material for: Geographical, temporal and individual factors influencing foraging behaviour and consistency in Australasian gannets
Source: R Soc Open Sci. 2020 May 27;7(5):181423. doi: 10.1098/rsos.181423 (PMC7277272; doi:10.1098/rsos.181423)
Supplement: Model selection results [file rsos181423supp2.zip › Supplementary Tables/Table S2 - RSOS-181423.R2.docx]

**Table S2** Average model coefficients and relative importance of variables included in top model set (∆AICc ≤ 4) explaining individual variation in Australasian gannets (*Morus serrator*) foraging metrics

| Foraging metric | Parameter | Estimate | SE | 5 %  CI | 95 %  CI | Relative  importance |
| --- | --- | --- | --- | --- | --- | --- |
| Maximum distance from the colony  (km) | (Intercept) | 4.06 | 0.40 | 3.42 | 4.72 | - |
|  | Colony (PE) | -0.81 | 0.09 | -0.96 | -0.66 | 1.00 |
|  | Breeding stage (INC) | 0.51 | 0.06 | 0.41 | 0.62 | 1.00 |
|  | Breeding stage (LCR) | -0.21 | 0.06 | -0.31 | -0.11 | 1.00 |
|  | Year (2015) | 0.14 | 0.05 | 0.06 | 0.21 | 1.00 |
|  | Sex (male) | -0.33 | 0.09 | -0.48 | -0.18 | 1.00 |
|  | WLI | 0.02 | 0.01 | -0.01 | 0.03 | 0.52 |
|  | BCI | 0.05 | 0.05 | -0.02 | 0.12 | 0.40 |
|  | BSI | 0.01 | 0.01 | -0.01 | 0.01 | 0.27 |
| Bearing (º) | (Intercept) | 213.12 | 27.04 | 167.93 | 257.68 | - |
|  | Colony (PE) | -72.44 | 6.23 | -82.76 | -62.13 | 1.00 |
|  | Breeding stage (INC) | -16.96 | 3.99 | -23.52 | -10.40 | 1.00 |
|  | Breeding stage (LCR) | 11.74 | 3.82 | 5.45 | 18.03 | 1.00 |
|  | Year (2015) | 13.69 | 3.32 | 8.19 | 19.12 | 1.00 |
|  | BSI | 0.01 | 0.01 | -0.01 | 0.03 | 0.51 |
|  | BCI | 3.38 | 2.99 | -1.55 | 8.30 | 0.39 |
|  | WLI | 0.26 | 0.73 | -0.95 | 1.45 | 0.26 |
|  | Sex (male) | 1.06 | 6.18 | -9.16 | 11.30 | 0.25 |
| Tortuosity Index | (Intercept) | 0.31 | 0.02 | 0.28 | 0.33 | - |
|  | Colony (PE) | -0.05 | 0.01 | -0.06 | -0.04 | 1.00 |
|  | Breeding stage (INC) | -0.05 | 0.01 | -0.06 | -0.04 | 1.00 |
|  | Breeding stage (LCR) | -0.01 | 0.01 | -0.01 | 0.01 | 1.00 |
|  | Year (2015) | 0.04 | 0.01 | 0.04 | 0.05 | 1.00 |
|  | WLI | 0.01 | 0.01 | -0.01 | 0.01 | 0.42 |
|  | BCI | 0.01 | 0.01 | -0.01 | 0.01 | 0.23 |
|  | Sex (male) | -0.01 | 0.01 | -0.01 | 0.01 | 0.23 |
|  | BSI | -0.01 | 0.01 | -0.01 | 0.01 | 0.22 |
| Mean VeDBA | (Intercept) | 0.83 | 0.02 | 0.79 | 0.86 | - |

|  | Breeding stage (INC) | -0.04 | 0.01 | -0.05 | -0.03 | 1.00 |
| --- | --- | --- | --- | --- | --- | --- |
|  | Breeding stage (LCR) | 0.01 | 0.01 | -0.01 | 0.01 | 1.00 |
|  | Sex (male) | 0.03 | 0.01 | 0.02 | 0.04 | 1.00 |
|  | Colony (PE) | 0.01 | 0.01 | -0.00 | 0.02 | 0.44 |
|  | Year (2015) | -0.01 | 0.01 | -0.01 | 0.01 | 0.22 |
|  | BSI | 0.01 | 0.01 | -0.01 | 0.01 | 0.16 |
|  | WLI | 0.01 | 0.01 | -0.01 | 0.01 | 0.16 |
|  | BCI | -0.01 | 0.01 | -0.01 | 0.01 | 0.16 |
| Number of dives | (Intercept) | 6.63 | 0.43 | 5.77 | 7.48 | - |
|  | Breeding stage (INC) | 1.53 | 0.18 | 1.24 | 1.83 | 1.00 |
|  | Breeding stage (LCR) | 0.28 | 0.15 | 0.02 | 0.53 | 1.00 |
|  | Sex (male) | -0.33 | 0.18 | -0.63 | -0.03 | 0.68 |
|  | Year (2015) | -0.21 | 0.16 | -0.47 | 0.05 | 0.44 |
|  | BSI | -0.01 | 0.01 | -0.01 | 0.01 | 0.22 |
|  | WLI | -0.01 | 0.02 | -0.04 | 0.03 | 0.16 |
|  | BCI | -0.01 | 0.09 | -0.15 | 0.15 | 0.16 |
|  | Colony (PE) | 0.01 | 0.18 | -0.30 | 0.30 | 0.16 |
