# Supplementary material for: Geographical, temporal and individual factors influencing foraging behaviour and consistency in Australasian gannets
Source: R Soc Open Sci. 2020 May 27;7(5):181423. doi: 10.1098/rsos.181423 (PMC7277272; doi:10.1098/rsos.181423)
Supplement: Model selection results [file rsos181423supp2.zip › Supplementary Tables/Table S3 - RSOS-181423.R2.docx]

**Table S3** Factors influencing individual variation (measured as the coefficient of variation within deployments for each foraging metric), at short- term scale (T-to-T) in Australasian gannets (*Morus serrator*). Most parsimonious models after model averaging and their corresponding estimated regression parameters are shown. The most parsimonious model was selected with using Akaike information criterion (AICc, ∆ < 4). Model variables: BCI: Body Condition Index; BSI: Body Size Index; WLI: Wing Length Index; stage: breeding stage; colony; year; sex

| Response | Most parsimonious model | Fixed effect | Estimate | SE | *t*-value | *P*-value |
| --- | --- | --- | --- | --- | --- | --- |
| Distance from colony (km)* | colony + year | (Intercept) | 0.28 | 0.01 | 24.32 | <0.0001 |
|  |  | Colony (PE) | -0.05 | 0.01 | -4.49 | <0.0001 |
|  |  | Year (2015) | -0.06 | 0.01 | -4.25 | <0.0001 |
| Bearing (º) | stage + year | (Intercept) | 1.60 | 0.04 | 37.19 | <0.0001 |
|  |  | Stage (INC) | -0.26 | 0.06 | -4.24 | <0.0001 |
|  |  | Stage (LCR) | 0.07 | 0.06 | 1.10 | 0.27 |
|  |  | Year 2015 | 0.11 | 0.05 | 2.06 | 0.04 |
| Tortuosity index | colony + WLI + year + sex | (Intercept) | 0.22 | 0.03 | 22.26 | <0.0001 |
|  |  | Colony (PE) | 0.04 | 0.01 | 3.68 | <0.0001 |
|  |  | Year (2015) | -0.04 | 0.01 | -3.96 | <0.0001 |
|  |  | Sex (male) | 0.02 | 0.01 | 2.54 | 0.01 |
|  |  | WLI | -0.01 | 0.01 | -2.70 | 0.01 |
|  |  | Sex (male) | 0.03 | 0.01 | 1.83 | 0.07 |
| Mean VeDBA (g)* | BSI | (Intercept) | 0.08 | 0.01 | 21.82 | <0.0001 |

|  |  | BSI | 0.01 | 0.01 | 2.70 | 0.01 |
| --- | --- | --- | --- | --- | --- | --- |
| Number of dives* | colony + stage + year | (Intercept) | 0.25 | 0.01 | 16.92 | <0.0001 |
|  |  | Colony (PE) | -0.04 | 0.01 | -2.66 | 0.01 |
|  |  | Year (2015) | -0.06 | 0.02 | -3.98 | 0.00 |
|  |  | Stage (INC) | 0.01 | 0.02 | 0.35 | 0.73 |
|  |  | Stage (LCR) | 0.06 | 0.02 | 3.57 | 0.001 |

*Transformed variable
